# Speech perception in patients submitted to cochlear reimplantation

**DOI:** 10.1590/2317-1782/20242023220en

**Published:** 2024-06-21

**Authors:** Lucas Bevilacqua Alves da Costa, Leticia Cristina Vicente, Leandra Tabanez do Nascimento Silva, Kátia Freitas Alvarenga, Manoel Henrique Salgado, Orozimbo Alves Costa, Rubens Brito

**Affiliations:** 1 Alfa Instituto de Comunicação e Audição – São Paulo (SP), Brasil.; 2 Universitário Planalto do Distrito Federal – Brasília (DF), Brasil.; 3 Centro de Pesquisa Audiológicas, Universidade de São Paulo – USP - Bauru (SP), Brasil.; 4 Departamento de Fonoaudiologia, Faculdade de Odontologia – FOB, Universidade de São Paulo – USP - Bauru (SP), Brasil.; 5 Departamento de Engenharia de Produção, Faculdade de Engenharia de Bauru, Universidade Estadual Paulista – UNESP - Bauru (SP), Brasil.; 6 Departamento de Otorrinolaringologia, Faculdade de Medicina – FM, Universidade de São Paulo – USP - São Paulo (SP), Brasil.

**Keywords:** Cochlear Implant, Equipment Failures, Auditory Perception, Review, Reoperation

## Abstract

**Purpose:**

To analyze the performance of auditory speech perception (PF) after cochlear implant (CI) replacement surgery and associations with age, times of use of the first CI, deprivation, recovery and use of the second device.

**Methods:**

The retrospective study analyzed the medical records of 68 participants reimplanted from 1990 to 2016, and evaluated with PF performance tests, considering as a reference, the greater auditory capacity identified during the use of the first CI. Also analyzed were: Etiology of hearing loss; the reasons for the reimplantation; device brands; age range; sex; affected ear; age at first implant; time of use of the first CI, deprivation, recovery and use of the second device. The analyzes followed with the Chi-Square and Spearman, Mann-Whitney and Kruskal-Wallis tests (CI=95%; p≤0.05; Software SPSS®.v22).

**Results:**

Most were children with hearing loss due to idiopathic causes and meningitis. Abrupt stoppage of operation was the most common cause for device replacement. Most cases recovered and maintained or continued to progress in PF after reimplantation. Adults have the worst recovery capacity when compared to children and adolescents. The PF capacity showed a significant association (p≤0.05) with: age at first implant; time of use of the first and second CI.

**Conclusion:**

Periodic programming and replacement of the device when indicated are fundamental for the maintenance of auditory functions. Being young and having longer use of implants represent advantages for the development of speech perception skills.

## INTRODUCTION

The impact of cochlear implants (CIs) on the rehabilitation and quality of life for those with hearing impairments is unquestionable. However, both users and professionals involved in the rehabilitation process need to be aware that a CI is an electronic device, and thus can experience issues such as sudden failure, known as “hard failures” or operational malfunction, called “soft failures”. The latter are generally harder to detect and are characterized by a gradual decline in the CI user’s audiological performance^(1,2)^.

Over time, a CI might fail, necessitating replacement of the internal component. Due to advances in this technology and the increased access to it, cochlear reimplantation has become increasingly common. Replacements are performed not only because of medical issues and device malfunction but also for technology upgrades^(2)^.

The rate of reimplantation among institutions varies from 5 to 10%^(1-3)^, and it is estimated that the risk of needing a replacement increases by about 1% with each year of CI use^(4)^. Thus, it can be stated that over a lifetime, a CI user will likely need to replace its internal component at some point. Although most studies have shown that reimplantation is a viable procedure and that patients generally maintain good audiological performance^(5-7)^, other studies have raised questions about possible impairments in auditory performance after the procedure^(3,4,6)^.

Considering this scenario, there are few national studies reporting the rate of reimplantation in institutions, and these studies do not discuss the performance of participants before and after the procedure^(8-10)^. Therefore, this study aimed to characterize the performance of auditory speech perception (SP) in individuals who underwent surgery to replace the internal component of the CI and determine possible associations with age, etiology of hearing loss, and times of use of the first and second CIs, auditory deprivation, and recovery of SP at a leading institution in Brazil.

## METHOD

### Study design

This is a longitudinal, retrospective, analytical study. It was conducted at the Cochlear Implant Section of the Hospital for Rehabilitation of Craniofacial Anomalies at the University of São Paulo (USP), Bauru Campus, state of São Paulo, Brazil. The study was approved by the Research Ethics Committee of the aforementioned institution under opinion no. 673.836 and followed the guidelines of the Helsinki Declaration. The authors signed a commitment agreement regarding the handling of information.

### Sample

The medical records of individuals who underwent a second surgical approach for the replacement of the CI after the diagnosis of SP capacity loss between 1990 and 2016 were analyzed. The protocol for CI replacement adopted by the institution includes the use of a new device from the same manufacturer.

### Inclusion and exclusion criteria

Inclusion criteria: individuals of all ages and both sexes, undergoing surgical procedures for CI replacement because of sudden cessation of operation, failure of the internal component, or for medical reasons such as electrode extrusion due to infections or intratemporal impairments.

Exclusion criteria: individuals with associated neurological impairments; those who did not effectively use the device; those who had poor adherence to follow-up and/or periodic assessments according to the protocols established by the institution; those with partial electrode insertion in the first surgery; bilateral CI users; those who had the electrode array removed and implanted in the opposite ear; those who were surgically reapproached for the implantation of a third CI (second replacement); those who only had the auditory skill of detection and did not acquire oral language.

### Assessment of auditory SP performance

The analysis of SP performance followed the protocol proposed by the institution’s Cochlear Implant Section, comprising the following tests: Word List, GlenDonald Auditory Screening Procedure (GASP), Hearing Test in Noise (HINT), and Sentence Recognition.

The Word List test is intended for hearing-impaired children aged 5 to 10 years. This test is conducted in a sound field using an audiometer, and involves the presentation of 20 disyllabic words, with a consonant-vowel-consonant-vowel (cvcv) syllabic structure, at an intensity of 70 dBHL, enabling the analysis of the consonant-vowel (cv) syllabic pattern, which is predominant in Portuguese. The children were instructed to repeat the words without using orofacial reading. The children’s utterances were phonetically transcribed by the evaluator, allowing for the recording of the results and calculation of the phoneme recognition score^(11)^.

For the GASP, tests 1, 3, 4, 5, and 6 were administered, assessing respectively: Ling sounds detection; vowel discrimination; duration discrimination of the vowel /a/; word recognition ability with the presentation of 12 words; comprehension ability of 10 sentences. The scores obtained in the tests are converted into a percentage of correct responses^(12)^.

The HINT, in its version translated and validated for Brazilian Portuguese, assesses SP in a sound field. It consists of 12 lists containing 20 sentences each, totaling 240 sentences available. The lists are presented randomly to ensure that the same sentences are not performed with the same individual. The Sentence Reception Threshold (SRT)/HINT is obtained when 50% of the sentences are correctly repeated in the presence of competing noise at a certain intensity. After presenting the sentence to the participant, the response will be accepted by the evaluator when all words are repeated correctly, only the definite and indefinite articles were changed, and words were added to the sentence without compromising the meaning^(13)^.

The Sentence Recognition test is composed of three lists of everyday sentences, presented in both a quiet environment and in the presence of competing noise. In an acoustic booth using an audiometer, a recorded list of 20 affirmative sentences in Portuguese is presented at an intensity of 60 dBHL to the patient, who is positioned at 0° azimuth on both vertical and horizontal planes relative to the speaker. Each sentence contains three to seven phonological words (keywords), totaling 100 keywords in each list presented without repetition. The score is calculated and transcribed as a percentage of correct responses. The same technique is used in the presence of competing noise (cocktail party) at a signal-to-noise ratio of +10 dBHL (S/N +10 dBHL)^(14)^.

All participants were evaluated throughout the use of the first CI (CI-1), during the deprivation period, which corresponds to the time when the need for replacement was diagnosed until the implantation of the second CI (CI-2), and during outpatient follow-ups with the CI-2.

### Reference data and comparison of SP capacity

The evaluation protocol used by the Cochlear Implant Section considers age, cognitive ability, and auditory skills acquired at the time of assessment. Thus, the same study participant may have been evaluated at various times during the follow-up using different assessment instruments, with the most suitable test being chosen based on the participant’s development and auditory capacity.

Because of the variability of the tests applied over time, this study considered the highest score obtained from the SP capacity evaluation tests through the use of the CI-1 as a reference. For comparison, the current SP score using the CI-2 was considered, obtained during the most recent multidisciplinary evaluation, and recorded in the medical record.

### Categorization of the SP capacity data

For the comparative analysis of SP capacity, a qualitative categorization method of performance results was adopted, consisting of the following categories:

- Did not recover: assigned to the participant whose SP capacity score during the use of the CI-2 did not reach the reference score identified during the use of the CI-1;
- Recovered and regressed: used when the CI-2 user achieved the reference score recorded during the use of the CI-1 but, subsequently, showed a decline in SP capacity;
- Recovered and maintained: category assigned to the individual who reached the reference score recorded during the use of CI-1 and maintained the SP capacity with the CI-2;
- Recovered and progressed: assigned to the participant who, after reimplantation, recovered SP capacity relative to the reference score and continued to develop auditory skills. This category refers to the best SP capacity observed during the last multidisciplinary evaluation using the CI-2.

### Epidemiological data and association variables

The following qualitative data were collected: sex; affected ear; etiology of hearing loss; brand of CI and reason for its replacement; age range (child or adult) – with children defined as individuals aged ≤12 years and adults as those aged >12 years.

Current SP qualitative data were organized dichotomously for inferential analysis using the following categories: 0 = did not recover (corresponding to individuals who did not recover and/or recovered and then regressed in SP capacity relative to the reference score); 1 = recovered (corresponding to individuals who recovered and maintained and/or those who recovered and continued to progress in SP capacity relative to the reference score).

The following quantitative variables were also analyzed: age of the participant when undergoing the implantation of CI-1 and when undergoing its replacement with CI-2; total use time of CI-1 until failure detection; deprivation time, which corresponds to the time elapsed since the failure of the CI-1 was detected until its replacement; recovery time, understood as the time elapsed until the CI reached the reference score (for those who recovered SP) or presented the highest score in auditory tests (for those who did not recover SP); total use time of CI-1 and CI-2. Time variables were described in months.

Clinical complications identified after device replacement were also recorded.

### Statistical analysis

The Chi-Square and Spearman tests were used to analyze the association between the variables, whereas the Mann-Whitney and Kruskal-Wallis tests were applied to compare categories. The tests were conducted at a 95% confidence interval and a significance threshold of 5% (*p*<0.05) using the SPSS^®^ v. 22 software.

## RESULTS

A total of 1,323 individuals underwent CI surgery, 84 of which were reimplanted, accounting for 6.4% of the surgical procedures. Of these, 16 participants were excluded from the study for not meeting the following exclusion criteria: poor adherence to treatment, rehabilitation, and follow-up appointments (n=10); ineffective use of the CI (n=1); the presence of bilateral CI (n=1); intratemporal complications after implantation (n=1); having not acquired oral language (n=1); being reimplanted more than once (n=1); partial insertion of electrodes because of ossification due to meningitis (n=1). Therefore, 68 individuals using unilateral CI and undergoing device replacement surgery participated in this study. Table 1 shows the epidemiological distribution by sex, affected ear, and etiology of hearing loss.

**Table 1:** Epidemiological profile data described for the sample

Variables	Children	Adults	N	%
Sex				
Female	18	18	36	52.9
Male	19	13	32	47.1
Ear				
Right	15	25	40	58.8
Left	22	6	28	41.2
Etiology of hearing loss				
Idiopathic	19	14	33	48.5
Infectious	10	13	23	33.8
Non-infectious	8	4	12	17.6

Considering the entire sample of children and adults (n=68), the participants' average age at the time of CI-1 implantation was 102.8 ±143.4 months. The mean usage time until device failure was detected was 73.3 ±63.2 months, and the deprivation time was 2.8±2.4 months, totaling an average usage time of 76.1±63.3 months for CI-1. Fifty (50) participants (73.5%) received their CI-1 before the age of six. The average age at the time of the CI-1 procedure was 37.4 ±11.8 months. At the time of the second approach for device replacement, participants had a mean age of 178.9 ±173.4 months. It took 6.5 ±7.1 months for the reimplanted individuals to regain hearing capacity at levels similar to those identified during the use of the CI-1, and the total usage time of the CI-2 up to the date of this study was 75.6 ±48.3 months. No clinical complications were identified after device replacement.

Table 2 presents a stratification by age range (children and adults) of the quantitative variables related to the age of the participants, device usage time, deprivation time, and usage time of the CI-1, as well as age at the time of device replacement, time elapsed for recovery, and total usage time of the CI-2.

**Table 2:** Stratification of quantitative time variables according to the age range of participants

Age range	Quantitative variables	Mean	SD	Median	Q1	Q3	Minimum	Maximum
Children								
	Age when CI-1 was implanted	37.2	14.3	34.0	27.0	45.5	14.0	84.0
	Usage time of CI-1 until failure	32.0	20.2	27.0	16.0	44.0	2.0	96.0
	Deprivation time	2.8	2.3	2.0	1.0	4.0	0.0	12.0
	Total CI-1 usage time	34.9	19.4	31.0	19.5	46.0	7.0	97.0
	Age when CI-2 was implanted	72.1	25.5	67.0	51.0	89.0	24.0	128.0
	Recovery time	7.2	6.9	4.0	3.0	12.5	0.0	33.0
	Total CI-2 usage time	94.7	37.6	104.0	65.0	123.5	10.0	143.0
**Adults**								
	Age when CI-1 was implanted	181.1	184.5	89.0	44.0	257.0	23.0	655.0
	Usage time of CI-1 until failure	122.5	61.9	124.0	78.0	157.0	8.0	271.0
	Deprivation time	2.7	2.6	2.0	1.0	4.0	1.0	10.0
	Total CI-1 usage time	125.3	62.6	128.0	79.0	158.0	9.0	280.0
	Age when CI-2 was implanted	306.3	188.5	225.0	170.0	480.0	144.0	854.0
	Recovery time	5.7	7.3	4.0	2.0	5.0	0.0	33.0
	Total CI-2 usage time	52.9	50.4	37.0	13.0	89.0	3.0	192.0

**Caption:** First cochlear implant (CI-1); second cochlear implant (CI-2); standard deviation (SD); first quartile (Q1); third quartile (Q3)

The reasons for device replacement were sudden cessation of operation, malfunction, and medical causes. There was a significant association (*p*<0.001) with the need for replacement with Med-El^®^ equipment, which corresponded to 69.1% of the sample, as per the distribution of the cases and the device brands provided by the service (Table 3).

**Table 3:** Distribution of reasons for replacement by brand and model of the internal component of the CI

Brand	N	%	Sudden cessation of operation	Operational failures	Medical reasons
Cochlear^®^	17	25	8	3	6
Med-El^®^	47	69.1	42	5	0
Advanced Bionics^®^	4	5.9	3	0	1
**Total**	68	100.0	53 (77.9%)	8 (11.8%)	7 (10.3%)

**Figure 1:**
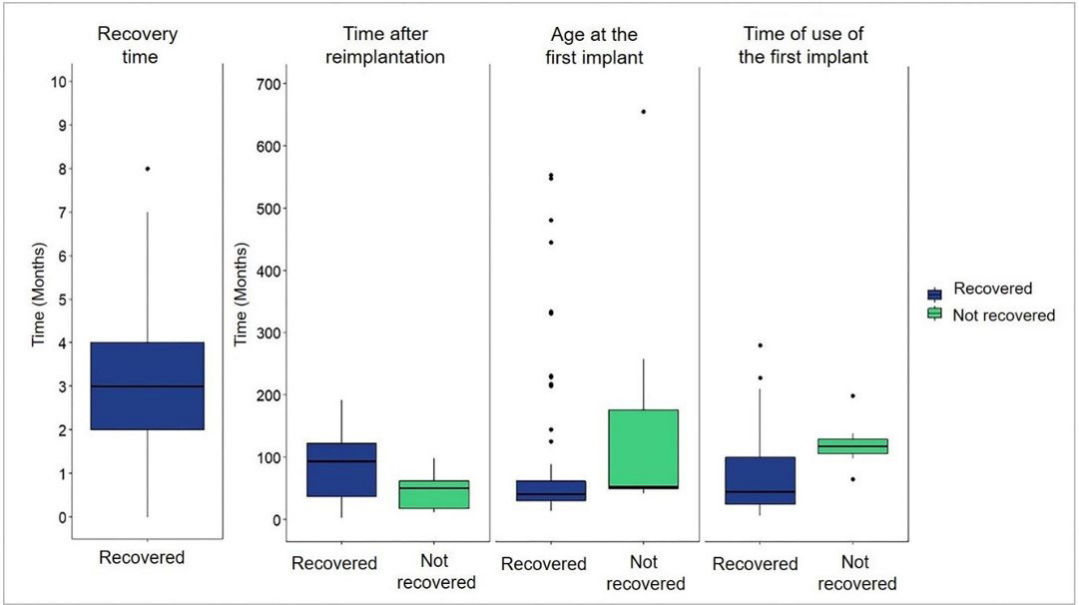
Distribution of the sample regarding SP recovery (recovered/ not recovered) in relation to the reference score recovery time, time after reimplantation, age at CI-1, and CI-1 usage time

All children recovered and either maintained or continued to progress in SP capacity with CI-2. For adults, the percentage of recovery of auditory skills was lower (77.4%). The analysis showed that 85.3% of the sample recovered, maintained, and/or continued progressing in auditory skills. The average recovery time was 6.6 ±7.3 months – a result that considered the period corresponding to the CI replacement surgery and the follow-up medical appointment, where the participant showed the best SP performance evaluated by specific tests. The medians, quartiles, and outliers are illustrated in Figure 1.

Table 4 shows the distribution of the participants who recovered their SP capacity according to recovery time. It can be noted that CI users who recover within four months after device replacement were younger when they received their first CI.

**Table 4:** Distribution of the sample that recovered and maintained and/or continued progressing in SP capacity relative to recovery time after device replacement and average ages at receiving the CI-1 and CI-2

Recovery time (months)	N	%	Average age at receiving CI-1	Average age at replacement with CI-2
0–4	36	62.1	73.4 ±104.2	145.7 ±125.3
5–8	8	13.8	90.0 ±158.6	123.8 ±157.3
≥9	14	24.1	99.5 ±120.9	175.6 ±169.6
**Total**	58	100	82.0 ±115.0	149.9 ±139.7

**Caption:** Recovery times and average ages are described in months. First cochlear implant (CI-1); second cochlear implant (CI-2)

For 14.7% (n=10) of the participants, satisfactory development was not identified, and all were adults. For these, there was an initial recovery followed by regression of auditory capacity or simply no recovery. The sample distribution according to the SP capacity identified with the use of the CI-2 is described in Table 5.

**Table 5:** Distribution of the sample according to SP capacity identified with the use of the second implant (CI-2) in relation to the reference score recorded during the use of the first implant (CI-1)

Age range	N	Recovered and progressed	Recovered and maintained	Recovered and regressed	Did not recover
	n	%	n	%	n
Children	37	34	91.9	3	8.1
Adults	31	14	45.2	7	22.6
**Total**	68	48	70.6	10	14.7

The 10 participants who did not satisfactorily recover auditory capacity presented the following etiologies of hearing loss: 2 idiopathic; 4 due to infectious causes associated with meningitis; 4 due to other non-infectious causes, such as cranioencephalic trauma, progressive hearing loss, neonatal hypoxia, and drug-induced ototoxicity. For these subjects whose SP capacity was not maintained or did not reach the reference score, the statistical analysis revealed that the older the individuals were when receiving the CI-1, the lower their SP capacity during the use of the CI-2 (Table 6).

**Table 6:** Analysis of the associations of quantitative variables with cases that did not show recovery or did not maintain the reference score for SP capacity

	Age at CI-1 implantation	Total CI-1 usage time	deprivation time	Recovery time	Total CI-2 usage time
p	0.008^*^	0.057	0.245	0.603	0.161

* *p*≤0.05: statistically significant

**Caption:** First cochlear implant (CI-1); second cochlear implant (CI-2)

The time of use of CI-1 and CI-2, as well as the age at the first surgery, showed significant association with the current best SP performance. The younger the individual received the CI-1 and the longer the time of use of CI-1 and CI-2, the higher the SP capacity score after device replacement (Table 7).

**Table 7:** Analysis of the associations of quantitative variables with cases that recovered the reference score or continued progressing in SP capacity

	Age when CI-1 was implanted	Total CI-1 usage time	Time until failure	Recovery time	Total CI-2usage time
p	0.003^*^	0.019*	0.940	0.787	0.019*

* *p≤*0.05: statistically significant

**Caption:** First cochlear implant (CI-1); second cochlear implant (CI-2)

The deprivation and recovery times did not show significant associations with SP performance (*p*>0.05).

## DISCUSSION

Currently, CI replacement is indicated because of failure of the internal component or for medical reasons, and it is recommended that a device from the same manufacturer be used. Studies have shown that device replacement can be safely performed with promising results regarding the recovery of SP^(15-17)^. However, these results pertain to a short follow-up period, raising questions about the long-term impact of this surgical intervention.

In this context, a study assessed the impact of reimplantation after one, two, and three years, and observed that there was an improvement in SP performance in 43.2% of the children evaluated, no change in performance in 40.5%, and worse performance in 16.2%^(7)^. Although the rate of failure is less significant, it is worth noting that there is a risk of worsening auditory skills and SP performance.

It is relevant to mention that the distribution of device failure occurrences according to brand in this study is an incidental finding, considering that many of these individuals were operated on in the 1990s – a time marked by the beginning of auditory habilitation and rehabilitation programs using CIs in Brazil when the importation of these devices followed a standard purchasing regulation that led to a large number of failures in devices of the same brand. A specific batch showed a change in the ceramic sealing of the internal component, which justified a recall promptly assumed by the company. In this study, 6.4% of users needed CI replacement over 25 years – a finding that agrees with the incidence of 1-10% described in the literature^(8-10,17-21)^.

In the cases studied, 89.7% of the CI replacements were indicated because of device failure, in contrast to 10.3% for medical reasons. These data corroborate findings from other studies that have demonstrated that internal component failure was the main cause of reimplantation^(20,22-25)^.

It should be emphasized that most individuals had access to the replacement of the internal component within less than four months; however, the deprivation time did not show a significant association with SP, and no other studies addressing the influence of deprivation time on the ability to recover hearing capacity were found.

In the cases studied, there were no surgical complications related to CI replacement, which differs from a previous study in which complications such as partial insertion of the electrodes was described^(26)^.

Despite the regression of SP capacity in three cases, 61 individuals (89.7%) recovered the reference score with the use of the CI-2, and only seven (10.3%) did not recover the reference hearing capacity. As described in the literature, in general, most individuals undergoing reimplantation manage to recover or improve their previous SP performance before reimplantation^(26-29)^.

Continuous progression of auditory skills with the use of the CI-2 was observed in 48 individuals (70.6%), and 34 (70.8%) of these were children in the period of developing more complex auditory skills, with no impact of the reimplant in this process. Additionally, 10 individuals (14.7%) did not recover the SP capacity according to the reference score, and three of these initially developed SP but subsequently regressed, and seven others did not even show signs of recovery during the rehabilitation period. Initially, the peculiarity of the results on auditory SP in these cases of reimplantation may be related to the modifications resulting from the removal and new insertion of the intracochlear electrode array, with an impact on the electrical stimulation of the cochlea^(30)^.

Clarck et al.^(30)^ reported that the electrode array can break and remain in the cochlea, but this does not prevent the placement of a new electrode. However, it is possible that the new electrode might be inserted partially and follow a different path than the previous one – conditions that can negatively influence the SP recovery process^(26,31)^. Nevertheless, these studies demonstrated that the audiological performance of the individual can be satisfactory in terms of maintaining effective oral communication, even after a second surgical intervention where the electrode array followed a different path in the cochlea.

Through routine radiological control in the intraoperative setting, it was ensured that, in this study, the insertion of the intracochlear electrodes was complete and without any compromise of this electrode array in all individuals undergoing reimplantation.

Overall, it was found that 73.5% of individuals received the CI-1 before the age of six. For the other 26.5% of the sample, the surgical approach after the age of six was justified by progressive or acquired hearing loss over life from various causes, adhering to assessment protocols and CI replacement indication criteria recommended in the literature.

Another relevant aspect is that only 25% of the individuals were reimplanted in the past two years preceding this study – a characteristic that favored a longer longitudinal time perspective, while other authors have conducted analyses within a period of 6 to 24 months after the CI replacement^(19,31,32)^.

Positively, the average recovery time for CIs was 6.6 months, and 58 individuals recovered and maintained, or even continued to develop SP capacity above the reference score recorded during the use of the CI-1, with 62.1% of these achieving this feat within four months after implantation of the CI-2. Some studies have reported that the average recovery time is around seven months, which corroborates our findings^(26,32)^. Another study showed improvement in SP capacity after three years of reimplantation, but these results were not statistically significant^(7)^. In this regard, it is crucial to advise individuals about the possibility of a delay in regaining previous SP performance.

The delay in the recovery of auditory SP after CI replacement is typically attributed to changes in the cochlea resulting from surgical manipulation. Another factor is that the new electrode array may stimulate a different group of ganglion cells, which may even differ in quantity (either a higher or lower number of cells) and, consequently, create a new pattern of stimulation to which the auditory cortex will need to adjust. Although there is no clear justification, the data show that individuals who received their first CI at a younger age exhibited earlier recovery of auditory skills. This confirms that the early years of life represent a window of opportunity for the habilitation and rehabilitation of auditory functions.

As a prominent result of this study, the age at which the device was received and the time of use of the CI-1 were significantly associated with SP recovery. Additionally, it can be stated that early surgical intervention did not interfere with the normal process of developing auditory skills and oral language. Thus, 48 individuals (70.6%) not only recovered after being reimplanted but also progressed in auditory SP, thereby performing more complex auditory tests.

Individuals who maintained their SP performance before CI replacement already had high scores on the applied tests and, for the most part, achieved the maximum possible score on the test. However, this group also included younger children, consequently performing tests that required only initial auditory skills. In these cases, the reason for non-development remains questionable, as the failure cannot be attributed solely to the reimplantation, since the treatment depends on numerous variables, among which are the quality of stimulation and the child’s cognitive ability.

It is noteworthy that three participants regressed in their auditory SP performance tests a few years after the CI replacement. This finding may be related to the causes of hearing loss, such as cranioencephalic trauma, meningitis, and progressive hearing loss without a defined cause, because of the progressive nature of these conditions. It is also worth noting that the regression of SP capacity was observed one year after the device was replaced – a fact that reinforces the need for continuous monitoring of CI users.

Another important aspect is that a history of meningitis was present in 56.5% of the cases of hearing capacity loss due to infectious causes. It was also present in four out of the 10 cases that did not recover SP capacity (40.0%) after the CI replacement. The incidence of this etiology in unsuccessful cases is higher than the 28.5% incidence reported in a previous study^(33)^. Particularly in cases of meningitis, there is damage and destruction of ganglion cells, as well as ossification and consequent obliteration of the tympanic and vestibular ramp lumen, precluding the physical insertion of an electrode capable of efficiently stimulating the cochlea in its different portions: basal, middle, and apical turns. It is important to emphasize that, even if an electrode array is inserted promptly before this obliteration, cochlear impairment does not cease upon its implantation.

In this context, it is observed that, in the periodic programming of the CI, the stimulation parameters in cases of meningitis are more variable, and may not provide the same pattern of stimulation again, negatively impacting auditory SP.

## CONCLUSION

The CI replacement surgery enables individuals with hearing impairments to regain their SP capacity and continue to progress in developing their auditory skills. Being younger at the time of receiving the CI-1 and having a longer usage time of both the CI-1 and CI-2 were factors associated with the recovery and progression of SP capacity.
