# Supplementary material for: Speech perception in patients submitted to cochlear reimplantation
Source: Codas. 2024 Jun 21;36(4):e20230220. doi: 10.1590/2317-1782/20242023220en (PMC11296668; doi:10.1590/2317-1782/20242023220en)
Supplement: Translation

# Translation: Percepção auditiva da fala após o reimplante coclear

**DOI:** 10.1590/2317-1782/20242023220pt

**Authors:** Lucas Bevilacqua Alves da Costa, Leticia Cristina Vicente, Leandra Tabanez do Nascimento Silva, Kátia Freitas Alvarenga, Manoel Henrique Salgado, Orozimbo Alves Costa, Rubens Brito

**Affiliations:** 1 Alfa Instituto de Comunicação e Audição – São Paulo (SP), Brasil.; 2 Universitário Planalto do Distrito Federal – Brasília (DF), Brasil.; 3 Centro de Pesquisa Audiológicas, Universidade de São Paulo – USP - Bauru (SP), Brasil.; 4 Departamento de Fonoaudiologia, Faculdade de Odontologia – FOB, Universidade de São Paulo – USP - Bauru (SP), Brasil.; 5 Departamento de Engenharia de Produção, Faculdade de Engenharia de Bauru, Universidade Estadual Paulista – UNESP - Bauru (SP), Brasil.; 6 Departamento de Otorrinolaringologia, Faculdade de Medicina – FM, Universidade de São Paulo – USP - São Paulo (SP), Brasil.

**Keywords:** Implante Coclear, Falha de Prótese, Percepção Auditiva, Revisão, Reoperação

## Abstract

**Objetivo:**

Analisar o desempenho da percepção auditiva da fala (PF) após cirurgia de substituição do implante coclear (IC) e identificar associações com a idade, tempos de uso dos dispositivos, privação e recuperação.

**Método:**

O estudo retrospectivo analisou os prontuários de 1990 a 2016 e considerou como referência o maior escore da capacidade auditiva identificada ao longo do uso do primeiro IC. Foram coletados dados epidemiológicos; etiologia; causas da substituição e marca dos dispositivos; classificação etária; idades no primeiro e segundo implante; tempos de utilização, privação e de recuperação da capacidade auditiva. Os dados foram avaliados por meio de testes estatísticos não paramétricos (IC=95%; p<0.05).

**Resultados:**

Foram avaliados 68 participantes (31 adultos e 37 crianças), sendo 52,9% do sexo feminino e as principais etiologias da perda auditiva foram: idiopática (48,5%), infecciosa (33,8%) e outras causas não infecciosas (17,6%). A idade média verificada na implantação do primeiro e do segundo IC, foram: 102±143,4 e 178,9±173,4 meses. Os tempos médios de uso do primeiro IC, privação, recuperação e uso do segundo IC, foram respectivamente: (76,1±63,3); (2,8±2,4); (6,5±7,1); (75,6±48,3) meses. A substituição foi motivada principalmente pela parada abrupta de funcionamento (77,9%) e 85,3% dos participantes recuperaram a PF, que esteve significativamente associada à idade no primeiro IC, e os tempos de utilização dos dispositivos (p<0.05).

**Conclusão:**

A maior parte dos indivíduos submetidos ao reimplante conseguem recuperar e/ou continuar o desenvolvimento das habilidades auditivas. A idade mais jovem e o tempo de uso dos dispositivos são fatores que influenciam na capacidade de recuperação da PF em reimplantados.

## INTRODUÇÃO

O impacto do implante coclear (IC) na reabilitação e qualidade de vida do deficiente auditivo é inquestionável. No entanto, é importante que os usuários e os profissionais envolvidos no processo de reabilitação, estejam atentos ao fato de que o IC é um dispositivo eletrônico e portanto, pode apresentar falhas como a parada abrupta denominada “*hard failure*” ou falhas no funcionamento denominadas “*soft failures*”, que em geral são mais difíceis de serem detectadas e se caracterizam pela queda gradual do desempenho audiológico do usuário de IC^(1,2)^.

A falha do IC ao longo do tempo pode resultar na necessidade de substituição do componente interno. Em virtude do avanço desta tecnologia e das possibilidades de acesso, o reimplante coclear tem sido uma realidade cada vez mais frequente. Além das questões médicas e das falhas no dispositivo, é relatado ainda na literatura a substituição do dispositivo por questões relativas ao *upgrade* de tecnologia^(2)^.

A taxa de reimplante entre as instituições varia de 5 a 10%^(1-3)^ e estima-se que o risco de substituição aumente em torno de 1% a cada ano de utilização do IC^(4)^. Desta forma, é possível afirmar que ao longo da vida, o usuário de IC possivelmente necessitará realizar a troca do dispositivo interno em algum momento. Neste contexto, embora a maior parte dos estudos demonstrem que o reimplante é um procedimento viável e que os pacientes, em geral, mantêm o bom desempenho audiológico^(5-7)^, outros estudos questionam possíveis comprometimentos no desempenho auditivo após o procedimento^(3,4,6)^.

Considerando este cenário, observa-se que há poucos estudos nacionais que relatam a taxa de reimplante nas instituições e estes estudos não mencionam o desempenho dos participantes antes e após o procedimento^(8-10)^. Sendo assim, este estudo teve como objetivo caracterizar o desempenho da percepção auditiva da fala (PF) dos indivíduos submetidos à cirurgia para substituição do componente interno do IC e determinar as possíveis associações com a idade, etiologia da perda auditiva, tempos de uso do primeiro e do segundo IC, de privação auditiva e de recuperação da percepção da fala em uma instituição de referência no Brasil.

## MÉTODO

### Desenho do estudo

Trata-se de um estudo longitudinal retrospectivo e analítico, aprovado por Comitê de Ética em Pesquisa, parecer número 673.836, e que seguiu a diretrizes da Declaração de Helsinque e os autores assinaram termo de compromisso de manuseio de informações. Foi realizado na Seção de Implante Coclear do Hospital de Reabilitação de Anomalias Craniofaciais, ambos da Universidade de São Paulo, Campus Bauru, do estado de São Paulo, Brasil.

### Amostra

Foram analisados os prontuários clínicos de 1990 a 2016, dos indivíduos submetidos a uma segunda reabordagem cirúrgica para substituição do implante coclear, após o diagnóstico da perda da capacidade de PF. A instituição adota como protocolo para a substituição do IC, a utilização de um novo dispositivo da mesma marca e fabricante.

### Critérios de inclusão e exclusão

Foram considerados como critérios de inclusão, os indivíduos de todas as idades e sexo, submetidos ao procedimento cirúrgico para substituição do implante coclear, devido parada abrupta ou falha do dispositivo interno ou causas médicas, tais como: Extrusão de eletrodos por infecções ou comprometimentos intratemporais.

Foram excluídos deste estudo indivíduos com comprometimentos neurológicos associados; aqueles que não fizeram uso efetivo do dispositivo; os que tiveram pouca adesão ao acompanhamento e/ou às avaliações periódicas por meio dos protocolos estabelecidos pela instituição; indivíduos com inserção parcial dos eletrodos na primeira cirurgia; indivíduos usuários de IC bilateral; indivíduos que tiveram o feixe de eletrodos extraído e foram implantados na orelha contralateral; indivíduos que foram reabordados cirurgicamente para implantação de um terceiro dispositivo (segunda substituição); indivíduos que apresentavam apenas a habilidade auditiva de detecção e não adquiriram a linguagem oral.

### Avaliação do desempenho da percepção auditiva da fala

A análise do desempenho da PF esteve orientada de acordo com o protocolo proposto pela Seção de Implante Coclear da instituição, composto pelos testes: Lista de palavras; *Glendonald Auditory Screening Procedure* *(GASP)*; *Hearing Test in Noise* *(HINT)*; teste de reconhecimento de sentenças.

O teste Lista de Palavras é indicado para crianças deficientes auditivas com idade entre 5 a 10 anos. O teste é aplicado em campo livre e com audiômetro, e constitui na apresentação de 20 palavras dissílabas com intensidade de 70 dBNA, com estrutura silábica composta por consoante-vogal-consoante-vogal (cvcv), permitindo a análise do padrão silábico consoante-vogal (cv), que é predominantemente na língua portuguesa. As crianças foram orientadas a repetirem as palavras sem fazer uso da leitura orofacial. As emissões das crianças foram transcritas foneticamente pelo avaliador, sendo possível registrar os resultados e realizar o escore de reconhecimento de fonemas^(11)^.

Para o *GASP* foram aplicadas as provas 1, 3, 4, 5 e 6, que avaliam respectivamente: Detecção dos sons do *Ling*; Discriminação de vogais; Discriminação de extensão da vogal /a/; Habilidade de reconhecimento com a apresentação de 12 palavras; Habilidade de compreensão de dez sentenças. As pontuações obtidas nas provas são convertidas em porcentagem de acertos^(12)^.

O *HINT* em sua versão traduzida e validada para o português brasileiro, avalia a percepção da fala em campo livre. É composto por 12 listas contendo 20 sentenças em cada, totalizando 240 sentenças disponíveis. As listas são apresentadas aleatoriamente, fazendo com que as mesmas sentenças não sejam realizadas com o mesmo indivíduo. O limiar de Recepção de Sentenças LRS/HINT é obtido quando 50% das sentenças são repetidas corretamente com ruído competitivo em determinada intensidade. Após a apresentação da sentença para o participante, a resposta será aceita pelo avaliador quando: Todas as palavras foram repetidas corretamente; apenas foi mudado o artigo definido e indefinido; Palavras foram adicionadas à sentença sem comprometer o sentido^(13)^.

O teste de reconhecimento de sentenças é composto por três listas de sentenças do cotidiano, que são apresentadas em ambiente silencioso e também na presença de ruído competitivo. Em cabina acústica por meio do audiômetro, é apresentada uma lista gravada com 20 sentenças afirmativas da língua portuguesa com intensidade de 60 dBNA, com o paciente posicionado a 0° azimute nos planos vertical e horizontal em relação à caixa. Cada sentença contém três a sete vocábulos fonológicos (palavras-chave), totalizando 100 palavras-chave em cada lista que são apresentadas sem repetição. O escore é calculado e transcrito em percentual de acertos. No ruído é utilizada a mesma técnica, com a presença de ruído competitivo (“*cocktail party”),* na relação sinal/ruído de +10 dBNA (S/R +10 dBNA)^(14)^.

Todos os participantes foram avaliados ao longo da utilização do primeiro IC (IC1); no período de privação, que corresponde ao momento que foi diagnosticada a necessidade de substituição, até o implante do segundo dispositivo coclear; nos seguimentos ambulatoriais com o segundo IC (IC2).

### Dados de referência e comparação da capacidade de percepção da fala (PF)

O protocolo de avaliação utilizado pela Seção de Implante Coclear, considera a faixa etária, capacidade cognitiva e as habilidades auditivas adquiridas no momento da avaliação. Deste modo, o mesmo participante do estudo pode ter sido avaliado em vários momentos do seguimento por meio de instrumentos de avaliação diferentes, sendo adotado o teste mais indicado e de acordo com o desenvolvimento e a capacidade auditiva do participante.

Em virtude desta variabilidade dos testes aplicados ao longo do tempo, o presente estudo considerou como referência o maior escore obtido nos testes de avaliação da capacidade da PF ao longo do tempo de uso do IC1. Como dado de comparação foi considerado o escore da PF atual com a utilização do IC2, obtido na avaliação multidisciplinar mais recente e registrada em prontuário.

### Categorização dos dados da capacidade de percepção da fala

Para a análise comparativa da capacidade da PF, foi adotado o método de categorização qualitativa dos resultados de desempenho, composta pelas seguintes categorias:

- Não recuperou: Atribuído ao participante cujo escore de capacidade da PF durante a utilização do IC2, não atingiu o escore de referência identificado durante o uso do IC1;
- Recuperou e regrediu: Utilizado quando o usuário do IC2 alcançou o escore de referência registrado durante o uso do IC1, mas posteriormente, apresentou um declínio do desempenho da PF.
- Recuperou e manteve: Categoria atribuída ao indivíduo que alcançou o escore de referência constatado durante a utilização do IC1 e manteve a capacidade da PF com o IC2;
- Recuperou e progrediu: Atribuído ao participante que após o reimplante, recuperou a capacidade da PF em relação ao escore de referência e ainda continuou desenvolvendo as habilidades auditivas. Esta categoria refere-se ao melhor desempenho da PF constatado durante a última avaliação multidisciplinar utilizando o IC2.

### Dados epidemiológicos e variáveis de associação

Foram coletados os seguintes dados qualitativos: sexo; orelha afetada; etiologia da perda auditiva; marca e motivo da substituição do IC; classificação etária (criança ou adulto), sendo considerado criança o indivíduo com idade ≤ 12 anos e adultos aqueles com idade superior a 12 anos.

Os dados qualitativos da PF atual foram organizados de forma dicotômica para a realização da análise inferencial, sendo utilizadas as seguintes categoria dicotômicas: 0 = não recuperou (corresponde aos indivíduos que não recuperaram e/ou recuperaram e em seguida regrediram em capacidade de PF, em relação ao escore de referência); 1 = recuperou (corresponde aos indivíduos que recuperaram e mantiveram e/ou àqueles que recuperaram e continuaram progredindo no desenvolvimento da PF, em relação ao escore de referência).

Também foram analisadas as seguintes variáveis quantitativas: idade do participante quando realizou o procedimento para implantação do IC1 e quando realizou a substituição pelo IC2; tempo total de utilização do IC1 até a detecção da falha; tempo de privação, que corresponde ao tempo decorrido desde a constatação da falha do IC1 até a substituição; tempo de recuperação, compreendido pelo tempo decorrido até o reimplantado coclear alcançar o escore de referência (para aqueles que recuperaram a PF) ou apresentar o maior escore aos testes auditivos (para aqueles que não recuperaram a PF); tempo total de utilização do IC1 e do IC2. As variáveis de tempo foram descritas em meses.

Complicações clínicas identificadas após a substituição do dispositivo, também foram registradas.

### Análise estatística

Para analisar a associação entre variáveis foi utilizado o Teste Qui-Quadrado e Spearman e para as comparações entre categorias, foram aplicados os testes de Mann-Whitney e Kruskal-Wallis. Os testes foram executados com intervalo de confiança de 95% e índice de significância p<0,05, com auxílio do software estatístico SPSS® versão 22.

## RESULTADOS

Foram constatados 1.323 indivíduos submetidos à cirurgia de IC, sendo 84 reimplantes, que corresponde a 6,4% dos procedimentos cirúrgicos. Destes, dezesseis participantes foram desconsiderados pois atendiam aos seguintes critérios de exclusão: Pouca adesão ao tratamento, reabilitação e consultas de acompanhamento (n=10); sem uso efetivo (n=1); Presença de IC bilateral (n=1); Complicações intratemporais após o implante (n=1); Não adquiriu linguagem oral (n=1); Foi reimplantando mais de uma vez (n=1); Inserção parcial dos eletrodos devido ossificação causada por meningite (n=1).

Participaram deste estudo 68 indivíduos usuários de IC unilateral e submetidos a cirurgia de substituição do dispositivo. A distribuição epidemiológica por sexo, orelha acometida e etiologia da perda auditiva, está descrita na Tabela 1.

**Tabela 1:** Dados descritivos do perfil epidemiológico da amostra

Variáveis		Crianças	Adultos	N	%
Sexo	Feminino	18	18	36	52,9
	Masculino	19	13	32	47,1
Orelha	Direita	15	25	40	58,8
	Esquerda	22	6	28	41,2
Etiologia da perda auditiva	Idiopática	19	14	33	48,5
	Infecciosa	10	13	23	33,8
	Não infecciosa	8	4	12	17,6

Considerando a totalidade da amostra e considerando crianças e adultos (N=68), os participantes tinham média de idade de 102,8±143,4 meses, quando foram operados para implantação do IC1. O tempo médio de utilização até a constatação da falha do dispositivo foi 73,3±63,2 e o tempo de privação foi de 2,8±2,4, totalizando o tempo médio de 76,1±63,3 meses de utilização do IC1.

Cinquenta participantes (73,5%) receberam o primeiro implante antes dos seis anos de idade. A média de idade destes no ato do primeiro procedimento para implante coclear era 37,4 ± 11,8 meses.

No momento da segunda abordagem para substituição do dispositivo, os participantes apresentavam idade média de 178,9 ± 173,4 meses. Os indivíduos reimplantados demoraram em média 6,5 ± 7,1 meses, para recuperar a capacidade auditiva aos níveis semelhantes aos identificados durante o uso do IC1 e o tempo total de utilização do IC2 até a data de realização deste estudo foi de 75,6 ± 48,3 meses. Não foi identificada nenhuma complicação clínica após a substituição do dispositivo.

Na Tabela 2 estão estratificadas por classificação etária (crianças e adultos), as variáveis quantitativas relacionadas à idade dos participantes, tempo de utilização do dispositivo, tempo de privação e de utilização do IC1, bem como, a idade no momento da substituição do dispositivo, tempo decorrido para a recuperação e o tempo total de utilização do IC2:

**Tabela 2:** Estratificação das variáveis quantitativas de tempo de acordo com a classificação etária dos participantes

Classificação etária	Variáveis quantitativas	Média	DP	Mediana	Q1	Q3	Mínimo	Máximo
Crianças	Idade quando implantou o IC1	37,2	14,3	34,0	27,0	45,5	14,0	84,0
	Tempo de uso do IC1 até a detecção da falha	32,0	20,2	27,0	16,0	44,0	2,0	96,0
	Tempo de privação	2,8	2,3	2,0	1,0	4,0	0,0	12,0
	Tempo total de uso do IC1	34,9	19,4	31,0	19,5	46,0	7,0	97,0
	Idade quando implantou o IC2	72,1	25,5	67,0	51,0	89,0	24,0	128,0
	Tempo de recuperação	7,2	6,9	4,0	3,0	12,5	0,0	33,0
	Tempo total de uso do IC2	94,7	37,6	104,0	65,0	123,5	10,0	143,0
Adultos	Idade quando implantou o IC1	181,1	184,5	89,0	44,0	257,0	23,0	655,0
	Tempo de uso do IC1 até a detecção da falha	122,5	61,9	124,0	78,0	157,0	8,0	271,0
	Tempo de privação	2,7	2,6	2,0	1,0	4,0	1,0	10,0
	Tempo total de uso do IC1	125,3	62,6	128,0	79,0	158,0	9,0	280,0
	Idade quando implantou o IC2	306,3	188,5	225,0	170,0	480,0	144,0	854,0
	Tempo de recuperação	5,7	7,3	4,0	2,0	5,0	0,0	33,0
	Tempo total de uso do IC2	52,9	50,4	37,0	13,0	89,0	3,0	192,0

**Legenda:** Primeiro implante coclear (IC1); Segundo implante coclear (IC2); Desvio padrão (DP); Primeiro quartil (Q1); Terceiro quartil (Q3)

Os motivos da substituição do dispositivo foram: Parada abrupta, falhas de funcionamento e causas médicas. Houve associação significativa (p<0,001), da necessidade de substituição com o equipamento da marca Med-El®, que corresponde a 69,1% da amostra, conforme distribuição da casuística e as marcas dos dispositivos fornecidos pelo serviço (Tabela 3).

**Tabela 3:** Distribuição das causas de substituição quanto à marca e modelo do componente interno do implante coclear

Marca	N	%	Parada abrupta	Falhas de funcionamento	Causas médicas
Cochlear^®^	*17*	*25*	8	3	6
Med-El^®^	*47*	*69,1*	42	5	0
Advanced Bionics^®^	*4*	*5,9*	3	0	1
Total	68	100,0	53 (77,9%)	8 (11,8%)	7 (10,3%)

Todas as crianças (100%) recuperaram e mantiveram ou continuaram a progredir quanto a capacidade de percepção da fala com o IC2. Quanto aos adultos o percentual de recuperação das habilidades auditivas foi menor, correspondendo a 77,4% dos adultos.

A análise demonstrou que 85,3% da amostra recuperou, manteve e/ou continuou progredindo nas habilidades auditivas. O tempo médio de recuperação foi 6,6 ± 7,3 meses, resultado que considerou o período correspondente à cirurgia de substituição do IC e a consulta médica de seguimento, na qual o participante apresentou o melhor desempenho de percepção da fala avaliado pelos testes específicos. As medianas, quartis e outliers, estão descritos na Figura 1.

**Figura 1:** Distribuição da amostra quanto à recuperação da percepção da fala (recuperou/não recuperou), em relação ao tempo de recuperação do escore de referência, tempo após o reimplante, idade ao implantar o primeiro IC e o tempo de utilização do primeiro IC

Na Tabela 4 os participantes que apresentaram recuperação da PF estão distribuídos de acordo com o tempo de recuperação. Nota-se que os usuários de IC que se recuperam em até quatro meses após a substituição do dispositivo, eram mais jovens quando receberam o primeiro IC:

**Tabela 4:** Distribuição da amostra que recuperou e manteve e/ou continuou progredindo no desenvolvimento da capacidade de percepção da fala em relação ao tempo de recuperação após substituição do dispositivo e médias de idade ao receber o primeiro e o segundo implante

Tempo de recuperação (meses)	N	%	Média de Idade ao receber o IC1	Média de idade no momento da substituição pelo IC2
0 a 4	*36*	*62,1*	73,4 ± 104,2	145,7 ± 125,3
5 a 8	*8*	*13,8*	90,0 ± 158,6	123,8 ± 157,3
≥ 9	*14*	*24,1*	99,5 ± 120,9	175,6 ± 169,6
Total	58	100,0	82,0 ± 115,0	149,9 ± 139,7

**Legenda:** Os tempos de recuperação e médias de idade estão descritas em meses. Primeiro implante coclear (IC1); segundo implante coclear (IC2)

Para 14,7% (n=10) dos participantes, não foi identificado desenvolvimento satisfatório e todos eram adultos. Para estes houve inicialmente recuperação com posterior regressão da capacidade auditiva ou simplesmente não recuperaram. A distribuição da amostra de acordo com a capacidade de PF identificada com o uso do segundo dispositivo, está descrita na Tabela 5.

**Tabela 5:** Distribuição da amostra de acordo com a capacidade de PF identificada com o uso do segundo implante (IC2), em relação ao escore de referência registrado durante o uso do primeiro implante (IC1)

Classificação etária	N	Recuperou e Progrediu		Recuperou e Manteve			Recuperou e Regrediu			Não Recuperou	
		n	%	n	%		n	%		n	%
Criança	37	34	91,9	3	8,1		0	0,0		0	0,0
Adulto	31	14	45,2	7	22,6		3	9,7		7	22,6
Total	68	48	70,6	10	14,7		3	4,4		7	10,3

Os dez participantes que não recuperaram a capacidade auditiva de forma satisfatória, apresentavam as seguintes etiologias da perda auditiva: 2 idiopáticas; 4 por causas infecciosas associadas a meningite; 4 por outras causas não infecciosas tais como trauma crânio-encefálico, deficiência auditiva progressiva, hipóxia neonatal e ototoxicidade induzida por fármacos. Para estes sujeitos cuja capacidade de PF não foi mantida ou não atingiu o escore de referência, a análise estatística revelou que quanto mais velhos são os indivíduos ao receber o IC1, menor a capacidade de PF durante a utilização do IC2 (Tabela 6).

**Tabela 6:** Análise das associações das variáveis quantitativas com os casos que não apresentaram recuperação ou não mantiveram o escore de referência da percepção da fala.

	Idade quando implantou o IC1	Tempo total de uso do IC1	Tempo de privação	Tempo de recuperação	Tempo total de uso do IC2
p	0,008*	0,057	0,245	0,603	0,161

* *p*≤0.05: estatisticamente significante

**Legenda:** Primeiro implante coclear (IC1); Segundo implante coclear (IC2)

O tempo de uso do IC1 e do IC2, bem como, a idade na primeira cirurgia, apresentaram associação significativa com o melhor desempenho atual da PF. Quanto mais jovem o indivíduo recebeu o IC1 e quanto maior o tempo de utilização do IC1 e do IC2, maior foi o escore da capacidade auditiva após a substituição do dispositivo (Tabela 7).

**Tabela 7:** Análise das associações das variáveis quantitativas com os casos que recuperaram o escore de referência ou continuaram progredindo na capacidade de percepção da fala.

	Idade quando implantou o IC1	Tempo total de uso do IC1	Tempo até a falha	Tempo de recuperação	Tempo total de uso do IC2
p	0,003*	0,019*	0,940	0,787	0,019*

* *p*≤0,05: estatisticamente significante

**Legenda:** Primeiro implante coclear (IC1); Segundo implante coclear (IC2)

O tempo de privação e de recuperação, não apresentaram associações significativas com a PF (p>0,05).

## DISCUSSÃO

Atualmente, a indicação de substituição do IC é mediante a falha do dispositivo interno ou por causas médicas, sendo preconizada a utilização do dispositivo da mesma marca/fabricante. Estudos têm demonstrado que a substituição do dispositivo pode ser realizada de forma segura e com resultados promissores quanto a recuperação da PF^(15-17)^. Entretanto, estes resultados referem-se a um curto período de acompanhamento, o que permite questionar se haveria impacto desta intervenção cirúrgica a longo prazo.

Neste contexto, um estudo avaliou o impacto do reimplante após um, dois e três anos, constatando que houve melhora na percepção de fala em 43,2% das crianças avaliadas, nenhuma mudança de desempenho em 40,5% e pior desempenho em 16,2%^(7)^. Apesar da taxa de insucesso ser menos representativa, é importante notar que há risco de piora das habilidades auditivas e desempenho da PF.

É relevante comentar que a distribuição da ocorrência de falha nos dispositivos de acordo com a marca neste estudo, é um achado ocasional, ao considerar que muitos desses indivíduos foram operados na década de 90, cuja época foi marcada pelo início dos programas de habilitação e reabilitação auditiva com IC no Brasil, cuja importação do dispositivo seguia uma normatização de compras, o que levou a um número elevado de falhas em aparelhos da mesma marca. Um lote específico apresentou alteração do selamento de cerâmica do componente interno, o que justificou um *recall* prontamente assumido pela empresa. No presente estudo 6,4% dos usuários necessitaram da substituição do IC ao longo de vinte e cinco anos, achado que está de acordo com a incidência de 1 a 10% descrita na literatura^(8-10,17-21)^.

Na casuística estudada, 89,7% dos casos a substituição do IC foi indicada devido a falha do aparelho, contra 10,3% motivado por razões médicas. Este dado contribui com outros estudos que demonstram que a falha do dispositivo interno é a causa principal de reimplante^(20,22-25)^.

É importante ressaltar que a maior parte dos indivíduos teve acesso a substituição do dispositivo interno em tempo inferior a quatro meses, contudo, o tempo de privação não apresentou associação significativa com a PF e não foram encontrados outros estudos que analisaram a influência do tempo de privação sobre a capacidade de recuperação da capacidade auditiva.

Na casuística estudada, não ocorreu nenhuma complicação cirúrgica relacionada a substituição do IC, o que difere de um estudo prévio a este, no qual foram descritas complicações como inserção parcial dos eletrodos^(26)^.

Apesar de ter sido constatada a regressão da capacidade de PF em três casos, sessenta e um indivíduos (89,7%) recuperaram o escore de referência com a utilização do IC2 e apenas sete (10,3%), não recuperaram a capacidade auditiva de referência. Conforme descrito na literatura, em geral, a maior parte dos indivíduos submetidos ao reimplante conseguem recuperar ou melhorar o desempenho anterior ao reimplante^(26-29)^.

A progressão contínua das habilidades auditivas com a utilização do IC2 foi observada em 48 indivíduos (70,6%), sendo que destes, 34 (70,8%) eram crianças e estavam no período de desenvolvimento de habilidades auditivas mais complexas, sem impacto do reimplante neste processo. Adicionalmente, dez indivíduos (14,7%) não recuperaram a capacidade de PF de acordo com o escore de referência, sendo que destes, três desenvolveram inicialmente a PF, porém regrediram posteriormente e outros sete, nem mesmo apresentaram sinais de recuperação da capacidade auditiva no período de reabilitação. A princípio, a peculiaridade dos resultados na percepção auditiva da fala nesses casos de reimplante pode estar relacionada com as modificações decorrentes da retirada e a nova inserção do feixe de eletrodos intracocleares, com reflexo na estimulação elétrica da cóclea^(30)^.

Clarck et al.^(30)^ relatam que o feixe de eletrodos pode se romper e permanecer na cóclea, entretanto este fato não impede a colocação de um novo eletrodo. Todavia, é possível que o novo eletrodo seja inserido parcialmente, além de ter um trajeto diferente do anterior, condições que podem influenciar negativamente no processo de recuperação da PF^(26,31)^. Contudo, estes estudos demonstram que o desempenho audiológico do indivíduo pode ser satisfatório, no sentido de manter uma comunicação oral efetiva, mesmo após uma segunda intervenção cirúrgica na qual o feixe de eletrodos seguiu um outro trajeto na cóclea.

Por meio do controle radiológico no intra-operatório realizado de rotina, é possível garantir que neste estudo, a inserção dos eletrodos intracocleares foi total e sem o comprometimento deste feixe de eletrodos em todos os indivíduos submetidos ao reimplante.

De maneira geral, constatou-se que 73,5% dos indivíduos receberam o IC1 antes dos seis anos de idade. Quanto aos outros 26,5% da amostra, a abordagem cirúrgica após os seis anos de idade foi justificada pela perda auditiva progressiva ou adquirida ao longo da vida por diferentes causas, respeitando os protocolos de avaliação e os critérios de indicação da substituição do IC, que são recomendados pela literatura.

Outro aspecto relevante é que, apenas 25% dos indivíduos foram reimplantados nos últimos dois anos que antecederam a realização deste estudo, característica que favoreceu uma perspectiva longitudinal de tempo mais prolongada, enquanto que outros autores realizaram uma análise em um período entre seis e vinte e quatro meses após a troca do IC^(19,31,32)^.

De forma positiva, a média de tempo de recuperação dos reimplantados foi de 6,6 meses e cinquenta e oito indivíduos recuperaram e mantiveram ou ainda, continuaram desenvolvendo a capacidade de PF acima do escore de referência registrado durante o uso do IC1, sendo que destes, 62,1% alcançaram este feito em até quatro meses após o implante do IC2. Alguns estudos relatam que o tempo médio para recuperação é em torno de sete meses, o que corrobora com nossos achados^(26,32)^. Um outro estudo demonstrou melhora na percepção de fala após três anos de reimplante, no entanto não houve significância estatística nesses resultados^(7)^. Neste sentido, é de fundamental importância a orientação ao indivíduo quanto a possibilidade de atraso na recuperação do desempenho anterior.

O atraso na recuperação do desempenho auditivo após a substituição do IC, normalmente é atribuído às modificações na cóclea decorrentes da manipulação cirúrgica. Outro fator é que o novo eletrodo pode estimular um grupo diferente de células ganglionares, que pode diferir inclusive em quantidade (maior ou menor número de células) e consequentemente, criar um novo padrão de estimulação com o qual o córtex auditivo necessitará se ajustar. Apesar de não haver uma justificativa evidente, os dados demonstram que os indivíduos que receberam o primeiro IC em idade mais jovem apresentaram a recuperação das habilidades auditivas de forma mais precoce, confirmando que os primeiros anos de vida são uma janela de oportunidade para a habilitação e reabilitação das funções auditivas.

Como resultado em destaque deste estudo, a idade ao receber o dispositivo e o tempo de utilização do IC1, foram fatores significativamente associados à recuperação da PF. Adicionalmente, é possível afirmar que a abordagem cirúrgica de forma precoce não interferiu negativamente no processo normal de desenvolvimento das habilidades auditivas e da linguagem oral. Assim, 48 indivíduos (70,6%) após serem reimplantados não apenas recuperaram, mas também evoluíram na percepção auditiva da fala, passando assim a realizar os testes auditivos de maior complexidade.

Quanto aos indivíduos que mantiveram o desempenho anterior a substituição do IC, já apresentavam escores elevados nos testes aplicados e em sua maioria, com escore máximo possível para o teste. Entretanto, neste grupo também se encontravam crianças com menor idade e consequentemente realizando testes que exigiam apenas habilidades auditivas iniciais. Nestes casos, fica questionável o motivo do não desenvolvimento, visto que o insucesso não pode ser atribuído ao reimplante exclusivamente, uma vez que o tratamento depende de inúmeras variáveis, dentre as quais, a qualidade da estimulação e cognitivo da criança.

Ressalta-se aqui o fato de que três participantes regrediram no desempenho aos testes de percepção auditiva da fala após alguns anos da substituição do IC. Este achado pode estar relacionado às causas da perda da capacidade auditiva, tais como o trauma crânio-encefálico, a meningite e a deficiência auditiva progressiva sem causa definida, devido ao caráter progressivo destas condições. Vale ressaltar que foi constatada a regressão da PF após um ano da substituição do dispositivo, fato que reforça a necessidade do acompanhamento contínuo ao usuário de IC.

Outro aspecto importante é que o histórico de meningite esteve presente em 56,5% dos casos de perda da capacidade auditiva por causas infecciosas. Também esteve presente em quatro, dos dez casos que não recuperaram a PF (40%) após a substituição do IC. A incidência desta etiologia nos casos de insucesso, é superior à incidência de 28,5% reportada em um estudo anterior^(33)^. Peculiarmente nos casos de meningite, observa-se o comprometimento e destruição das células ganglionares, além da ossificação e consequente obliteração da luz da rampa timpânica e vestibular, impedindo que seja fisicamente inviável a inserção de um eletrodo capaz de estimular de forma eficiente a cóclea em suas diferentes porções, espira basal, média e apical. É importante destacar que mesmo que haja a inserção de eletrodos em tempo hábil, antes dessa obliteração, o comprometimento coclear não cessa ao realizar a implantação de um eletrodo.

Neste contexto, observa-se que na programação periódica do implante coclear, os parâmetros de estimulação nos casos de Meningite são mais variáveis e podem não propiciar novamente o mesmo padrão de estimulação, com impacto negativo na percepção auditiva da fala.

## CONCLUSÃO

A cirurgia de substituição do IC permite que os indivíduos com deficiência auditiva, recuperem a capacidade de percepção da fala e continuem a progredir no desenvolvimento das habilidades auditivas. A menor idade ao receber o primeiro dispositivo e o maior tempo de utilização do primeiro e do segundo IC, foram aspectos associados à recuperação e progressão das habilidades de percepção da fala.
